# Draft Genome Sequence of a Stable Mucoid Strain of *Pseudomonas aeruginosa* PAO581 with a *mucA25* Mutation

**DOI:** 10.1128/genomeA.00834-13

**Published:** 2013-10-10

**Authors:** Yeshi Yin, T. Ryan Withers, John R. W. Govan, Shannon L. Johnson, Hongwei D. Yu

**Affiliations:** Departments of Biochemistry and Microbiology, Joan C. Edwards School of Medicine at Marshall University, Huntington, West Virginia, USAa; Department of Pediatrics, Joan C. Edwards School of Medicine at Marshall University, Huntington, West Virginia, USAb; Progenesis Technologies, LLC, Huntington, West Virginia, USAc; Cystic Fibrosis Laboratory, University of Edinburgh Medical School, Chancellor’s Building, Edinburgh, United Kingdomd; Genome Science Group (B6), Los Alamos National Laboratory, Los Alamos, New Mexico, USAe

## Abstract

A mutation in the *mucA* gene, which encodes a negative regulator of alginate production in *Pseudomonas aeruginosa*, is the main mechanism underlying the conversion to mucoidy in clinical isolates from patients with cystic fibrosis (CF). Here, we announce the draft genome sequence of the stable alginate-overproducing mucoid strain *P. aeruginosa* PAO581 with a *mucA25* mutation, a derivative from the nonmucoid strains *P. aeruginosa* PAO381 and PAO1.

## GENOME ANNOUNCEMENT

Alginate overproduction in *Pseudomonas aeruginosa*, also termed mucoidy, is a poor prognostic marker in patients with cystic fibrosis (CF). Alginate regulation has been extensively studied in *P. aeruginosa* (1–3). Two mechanisms for the conversion to mucoidy have been elucidated. The first one is through a mutation in the *mucA* gene (4), and the second is through the activation of intramembrane proteolysis leading to the degradation of MucA (3). Both mechanisms converge to release AlgU from sequestration by MucA, subsequently initiating transcription of the *algD* operon (5, 6). Here, we sequenced the genome of a stable mucoid variant, *P. aeruginosa* PAO581 (*mucA25*), which was isolated *in vitro* following the incubation of the nonmucoid *P. aeruginosa* strain PAO381 with phage E79 (7). PAO381 is a derivative of the progenitor strain *P. aeruginosa* PAO1, with a streptomycin resistance and FP2 marker (8).

The genomic DNA of PAO581, which was extracted by cetyltrimethylammonium bromide (CTAB)-NaCl and phenol-chloroform-isoamyl alcohol, was sent to Cofactor Genomics (St. Louis, MO) for whole-genome sequencing. Paired-end sequencing libraries were generated according to the vendor protocols (Illumina, San Diego, CA). The genome sequencing was performed on an Illumina GAIIx. A total of 26,126,768 raw reads and 2,612,676,800 bp were obtained. The sequence data were generated and assembled using the Illumina Pipeline version SCS 2.8.0 based on paired-end tags with OLB 1.8.0. The sequences were aligned and annotated according to the reference genome of *P. aeruginosa* PAO1 (GenBank accession no. NC_2516.2) using the Novocraft Novoalign version 2.07.10 software package. Further analysis of the genome was performed using SAMtools version 01.16a for the generation of pileup after sorting and removing duplicate reads. The analysis pipeline software was developed by Cofactor Genomics, and all specifics regarding the aligner algorithms can be obtained from the Novocraft website. The coverage of the generated sequences is 199× the reference genome. The number of base pairs saturated at ≥8× is 6,003,484 (95.83%), and the number of base pairs saturated at <8× is 260,920 (4.17%). The genome was annotated and prepared for submission using an Ergatis-based workflow with manual correction.

Analysis of single nucleotide polymorphisms (SNPs) and indels showed that a total of 44 heterozygous mutations (30 indels and 14 SNPs) (0.3 ≤ count ratio ≤ 0.8) and 22 homozygous SNPs (count ratio, >0.8) were identified in PAO581 in comparison to the reference genome of PAO1. The count ratio is derived by the number of times the reference base is observed divided by coverage at this base, including all matches and mismatches. Among the indels, 16 were found in the intergenic regions, and 14 were found inside genes causing a frameshift. Among the homozygous SNPs, 17 SNPs were distributed in 15 genes causing nonsynonymous or frameshift mutations, and 5 SNPs were in intergenic regions. Among the mutant genes, only *mucA* (3) and *clpP* (9) have been previously reported to be involved in alginate regulation.

### Nucleotide sequence accession number.

The draft genome sequence of the stable alginate-overproducing mucoid strain PAO581 has been deposited in GenBank under the accession no. CP006705.
